# Antiherpes Activity and Skin/Mucosa Distribution of Flavonoids from *Achyrocline satureioides* Extract Incorporated into Topical Nanoemulsions

**DOI:** 10.1155/2015/238010

**Published:** 2015-05-25

**Authors:** Juliana Bidone, Débora Fretes Argenta, Jadel Kratz, Letícia Ferreira Pettenuzzo, Ana Paula Horn, Letícia Scherer Koester, Valquíria Linck Bassani, Claudia Maria Oliveira Simões, Helder Ferreira Teixeira

**Affiliations:** ^1^Programa de Pós-Graduação em Ciências Farmacêuticas da Universidade Federal do Rio Grande do Sul (UFRGS), Avenida Ipiranga 2752, 90610-000 Porto Alegre, RS, Brazil; ^2^Programa de Pós-Graduação em Farmácia da Universidade Federal de Santa Catarina (UFSC), Rua Delfino Conti s/n, 88040-970 Florianópolis, SC, Brazil; ^3^Programa de Pós-Graduação em Ciências Biológicas (Bioquímica) da Universidade Federal do Rio Grande do Sul (UFRGS), Rua Ramiro Barcelos 2600, 90035-003 Porto Alegre, RS, Brazil; ^4^Programa de Pós-Graduação em Ciências Fisiológicas: Fisiologia Animal Comparada da Universidade Federal de Rio Grande (FURG), Avenida Itália Km 8, 96203-900 Rio Grande, RS, Brazil

## Abstract

This study investigated the inhibitory effects of *Achyrocline satureioides* extract (ASE) incorporated into a topical nanoemulsion on Herpes Simplex Virus type 1 (HSV-1/KOS strain) replication, as well as the distribution of the main ASE flavonoids (quercetin, luteolin, and 3-*O*-methylquercetin) in porcine skin and mucosa. The ASE-loaded nanoemulsion showed more pronounced effects against HSV-1 replication when compared to the ASE or pure quercetin, as determined by the viral plaque number reduction assay. All flavonoids were detected in the skin epidermis (2.2 *µ*g/cm^2^) and the mucosa upper layers (3.0 *µ*g/cm^2^) from ASE-loaded nanoemulsion until 8 h after topical application. A higher amount of flavonoids was detected when these tissues were impaired, especially in deeper mucosa layers (up to 7-fold). Flavonoids were detected in the receptor fluid only when the mucosa was injured. Such results were supported by confocal microscopy images. Overall, these findings suggest that the tested ASE-loaded nanoemulsion has potential to be used topically for herpes infections.

## 1. Introduction

Oral herpes is an infection of the lips and surrounding areas caused mainly by Herpes Simplex Virus type 1 (HSV-1). This infection has emerged in recent years, especially in immunocompromised hosts, such as in AIDS and transplanted patients. Herpetic infections may result from primary contact with the virus or reactivation of a latent infection. Several drugs are currently available for the treatment of HSV-1 infections, especially nucleoside analogues. However, the emergence of resistant viral strains to these molecules has stimulated the search for novel antiherpes drugs [1, 2].

**Figure 1:**
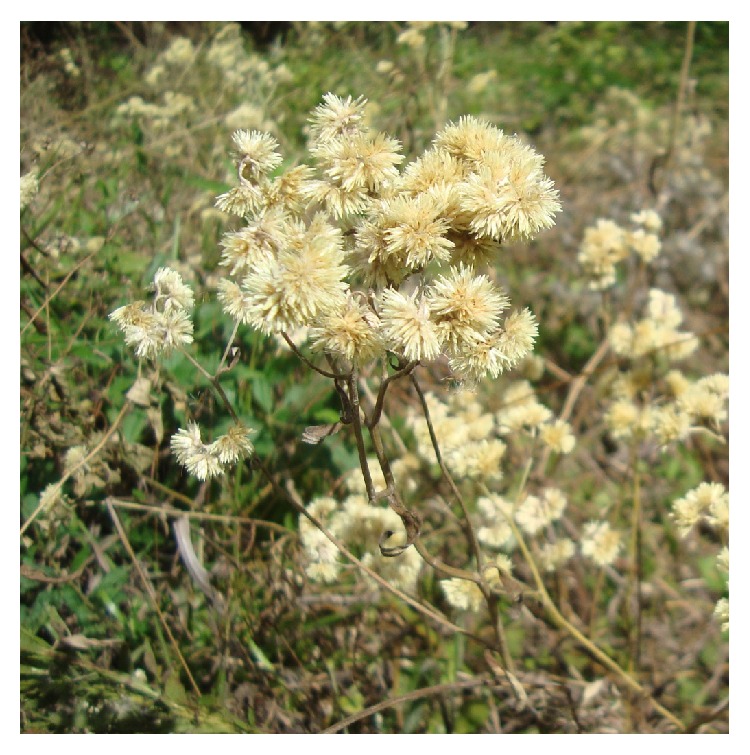
Inflorescences of* Achyrocline satureioides* (Lam.) DC., Asteraceae.

Isolated compounds, fractions, or even crude extracts obtained from medicinal plants have been considered as a promising strategy for antiherpes drug research [1–3]. In this way, extracts of* Achyrocline satureioides* (Lam.) DC., Asteraceae (Figure 1), a medicinal plant whose aerial parts are widely used in folk medicine [4], have shown antiherpes activity against HSV-1 (KOS strain) [5]. This activity was detected between the second and ninth hour of HSV-1 replication cycle, probably indicating a perturbation in the late stages of this cycle. Such activity was mainly related to the presence of flavonoid aglycones, which are quercetin (QCT), luteolin (LUT), and 3-*O*-methylquercetin (3-*O*-MQ).

We also have recently described the simultaneous incorporation of these poorly soluble flavonoids of* Achyrocline satureioides* extract into nanoemulsions by means of spontaneous emulsification intended for topical use [6]. Such a procedure proved to be able to efficiently incorporate the main flavonoids from the hydroethanolic extract in monodisperse nanoemulsions (200 nm range) consisting of a medium chain triglycerides oil core stabilized by a binary mixture of surfactants (egg lecithin and polysorbate 80). Flavonoids seem to be located in the oil phase of nanoemulsions since they were not detected in the aqueous phase after separation on ultrafiltration membranes. The 3-*O*-MQ release rate was influenced by the extract content in the nanoemulsion with approximately 90% released after 8 h of kinetics. The first-order model provided the most satisfactory fitting of the release data from extract-nanoemulsions [6].

Following up on these results, this study investigated the potential of this formulation as a delivery system for the* Achyrocline satureioides* extract (ASE) intended for antiherpes topical application. Thus, the activity of the ASE-loaded nanoemulsion against HSV-1 and the distribution of the main flavonoids in the skin and mucosa layers, which are the main sites of herpetic infections [7], were investigated. Since the local treatment of oral herpes includes applying the medication before and/or after the blisters appear, flavonoid distribution was also evaluated in the impaired epithelial barrier to gain a better insight on the effect of the integrity of these tissues on flavonoid retention.

## 2. Materials and Methods

### 2.1. Materials

The* Achyrocline satureioides* plant (voucher specimen: number 308) was obtained from CPQBA-UNICAMP (São Paulo, Brazil). Egg-lecithin (Lipoid E-80) and medium chain triglycerides (MCT) were purchased from Lipoid GmbH (Ludwigshafen, Germany). Polysorbate 80 and vitamin E were supplied from Vetec Química Fina Ltda. (Rio de Janeiro, Brazil) and Alpha Química (Porto Alegre, Brazil), respectively. Quercetin, luteolin, and 3-*O*-methylquercetin standards were obtained from Sigma (Steinheim, Germany), Alfa Aesar (Lancashire, United Kingdom), and Extrasynthese (Genay, France), respectively. Nile Red was purchased from Sigma (Steinheim, Germany). Porcine ears and esophagus were gently donated by Ouro do Sul Ltda. (Harmonia, Brazil). All solvents used were HPLC grade: methanol (J.T. Baker, Center Valley, USA), acetonitrile (Tedia Brasil, Rio de Janeiro, Brazil), and phosphoric acid (Merck, Darmstadt, Germany).

### 2.2. Chromatographic Conditions

QCT, LUT, and 3-*O*-MQ content into crude extract, nanoemulsions, and skin/mucosa after permeation studies were determined using a liquid chromatography equipment Shimadzu LC-10A, equipped with LC-10AD pump, CBM-10A system controller, SIL-10A autosampler, SPD-20AV UV/vis detector (set at 362 nm), and LC Solution software. The chromatographic system was composed by a Synergi Polar-RP 150 × 4.6 mm i.d., 4 *μ*m (Phenomenex, Torrance, CA) column protected by precolumn packed with silica C18 Phenomenex (150 *μ*m, 140 Å), temperature system of 30 ± 1°C, isocratic flux of 0.8 mL/min, and 20 *μ*L as injection volume. The mobile phase consisted of methanol : 0.16 M phosphoric acid : acetonitrile (46 : 44 : 10, v/v/v) and the samples were diluted in methanol : phosphoric acid 16 mM (50 : 50, v/v) before analyses. The analytical method was previously validated for the determination of QCT, LUT, and 3-*O*-MQ in ethanolic extract and nanoemulsion [8], demonstrating to be specific, linear (0.25 to 10 *µ*g/mL), precise, and accurate. In this paper, the revalidation of the analytical method was performed in terms of specificity and recovery of QCT, LUT, and 3-*O*-MQ from porcine ear skin and porcine esophageal mucosa. The limits considered acceptable for the evaluated parameters are in accordance with the “The Guidance for Industry: Bioanalytical Method Validation” (FDA).

### 2.3. Preparation of the Nanoemulsions

In a first step, an* Achyrocline satureioides* extract (ASE) was obtained from aerial parts of* Achyrocline satureioides* by means of maceration process in ethanol 80% (v/v) over eight days, using plant : solvent proportion of 7.5% (w/v). The extract was filtered before use.

The preparation of the nanoemulsions was carried out by spontaneous emulsification procedure, according to Bidone et al. [6]. Briefly, the compounds of the oil core, medium chain triglycerides (7.5 wt.%), egg-lecithin (2.0 wt.%), and vitamin E (0.5 wt.%) were dissolved in ethanol. 9.5 mL of ASE was added to this ethanol phase (that corresponds to 1.0% of dried residue). Then, this organic phase was poured into the water phase composed of polysorbate 80 (1.0% wt.%) and water under moderate and constant magnetic stirring. The ethanol/water ratio used was 30 : 60 (v/v). Afterwards, the formulation was concentrated to 10 mL by evaporation under reduced pressure at 40°C. Quercetin-loaded nanoemulsions (QCT-NE) at 300 *µ*g/mL of flavonoid and nanoemulsions without ASE (Blank-NE) were prepared as control formulations. All formulations were prepared in triplicate.

### 2.4. Flavonoids Content in ASE and Nanoemulsions

For the determination of the flavonoid content in ASE, a solution was prepared from crude extract (1 mL of extract into 10 mL hydroethanolic solution), being then diluted (15 *µ*L/mL in methanol : 16 mM phosphoric acid (50 : 50, v/v)) and analyzed as described in Section 2.2. The results were expressed as QCT, LUT, or 3-*O*-MQ concentration (*µ*g) per 10 mg of extract dried residue (considering that 1.0% of dried residue, or 10 mg, was incorporated in 1 mL of nanoemulsion) [6]. The crude* A. satureioides* ethanolic extract has 1.21% of dried residue.

The total flavonoids content was determined after the dilution of nanoemulsions in methanol and in a methanol : phosphoric acid 16 mM (50 : 50, v/v) solution. To estimate the flavonoids association, nanoemulsions were directly added to ultrafiltration membranes (100,000 Da cutoff, Ultrafree; Merck Millipore) and centrifuged at 10,000 rpm for 50 minutes. Free flavonoids were estimated in the ultrafiltrate. The association efficiency (%) was estimated by the difference between the total and free-flavonoids concentrations. All results were presented as the mean of three analyses.

### 2.5. Characterization of the Nanoemulsions

Nanoemulsions were diluted in water and the mean droplet size and the polydispersity index were measured by photon correlation spectroscopy (PCS) at 25°C. The determination of the *ζ*-potential of nanoemulsions was performed by electrophoretic mobility, after dilution in 1 mM NaCl. Analyses were carried out in triplicate using a Zetasizer Nano-ZS90 (Malvern Instruments, England). The viscosity was determined by capillary viscometry in Ostwald viscometer, using a bath at 20°C and a number 2 capillary.

### 2.6. Permeation/Retention Assay in Intact and Impaired Tissues

The permeation/penetration assay was carried out by method of the Franz type diffusion cells using porcine ear skin and esophageal mucosa. Throughout the experiment time the system was kept under a controlled temperature (32 ± 1°C) and constant stirring. Skin or esophageal pieces were set between the donor and receptor compartments of the Franz cell, on a surface area of 2.54 cm^2^. The receptor compartment was supplied with a mixture of PBS : ethanol (70 : 30). 500 *µ*L of nanoemulsion containing* Achyrocline satureioides* extract was then applied on the donor compartment. After eight hours, an aliquot of the fluid receptor was withdrawn and the skin or mucosa was removed from the cell.

The pieces were cleaned with PBS and cut in small pieces. The flavonoids were extracted with methanol using an ultrasonic bath for 40 minutes. For the evaluation of impaired skin, tape stripping was performed by using 60 tapes (Scoth 750 tape, 3 M) before the experiment. In turn, the impaired mucosa was obtained by removing the superficial epithelium layer with a scalpel.

After permeation assay, skin and esophageal mucosa were paraffin embedded and slices (6 *µ*m thick) were cut and stained with hematoxylin-eosin to be analyzed by optical microscopy. Moreover, a confocal microscopic Olympus FluoView 1000 was used to evaluate the tissues after permeation/retention assay using nanoemulsion containing Nile red as oil core dye. The fluorescence intensity was determined using ImageJ software.

### 2.7. *In Vitro* Assays

The cytotoxicity of QCT and of the hydroethanolic extract of* A. satureioides* and their nanoemulsions on healthy cells was evaluated by tetrazolium-based colorimetric assay (MTT). Vero cells were grown in minimum essential medium (MEM) supplemented with 10% fetal bovine serum and PSA (penicillin, streptomycin, and amphotericin), at 37°C and 5% CO_2_. These cells were trypsinized, distributed along a plate (100 *μ*L/cavity), and incubated by 24 hours. Then, the samples were diluted in MEM and added to Vero cell monolayers. After incubation for 36 h, the concentration of each sample that reduced cell viability by 50%, that is, the cytotoxic concentration for 50% of the cells (CC_50_), was calculated. Cells exposed to MEM without samples or exposed to blank-NE were used as controls.

For antiherpes activity evaluation, the viral plaque number reduction assay was performed as described by Argenta et al. [9]. Firstly, Vero cell monolayers were infected with HSV-1 suspension (100 PFU/well), except in the cellular control. After 1 h at 37°C and 5% CO_2_, cells were washed with PBS and different concentrations of the samples were added and then incubated for 36 h. Cells were then fixed and stained with naphthol blue black for viral plaques counting. The inhibitory concentration (IC_50_) was defined as the concentration that inhibited 50% of viral plaque number when compared to untreated controls. The selectivity index (SI = CC_50_/IC_50_) was also determined for each sample.

### 2.8. Statistical Analysis

The statistical analysis of the results was performed by one-way analysis of variance (ANOVA) and Tukey's test using GraphPad Prism 5 software. The level of significance chose was less than 0.05.

## 3. Results and Discussion

As stated before, we have recently described the simultaneous incorporation of the main poorly soluble flavonoids (QCT, LUT, and 3-*O*-MQ) from the ethanolic extract of* Achyrocline satureioides* into nanoemulsions [6], intended for the local treatment of HSV-1 infection.  Table 1 summarizes the physicochemical characteristics of this formulation. The procedure yielded monodisperse nanoemulsions (polydispersity index < 0.1) with a mean droplet size in the 200 nm range. The association efficiency of flavonoids with the oil core of nanoemulsions was close to 100% as none of them was detected in the external aqueous phase of nanoemulsions after separation using ultrafiltration membranes. Moreover, the amount of flavonoids in the ASE-loaded nanoemulsions remained similar to that detected in ASE, indicating that flavonoids were not lost during spontaneous emulsification procedure (Table 2). Such results may be attributed to the affinity of flavonoid aglycones for the oil core of nanoemulsions (composed of triglycerides/egg lecithin) due to their poor water solubility. In turn, *ζ*-potential increased (in modulus) from approximately −20 mV to −43 mV, suggesting that other extract compounds, such as phenolic acids, may be adsorbed at the oil-water interface. These results are in accordance with our previous study [6], which showed that experimental conditions were well controlled.

The incorporation of* Achyrocline satureioides* extract into a nanoemulsion significantly reduced (*P* < 0.05) the concentration that inhibited 50% of HSV-1 plaque number and the IC_50_ value decreased from 14.07 to 1.40 *µ*g/mL (Table 3). This result suggests that the nanoemulsions tested could enhance intracellular flavonoids uptake. Previous works have reported an increase of flavonoids cell uptake when they were incorporated into nanocarriers [9, 10]. This activity was also significantly higher (*P* < 0.05) than that observed for pure quercetin (9.5 *µ*g/mL), suggesting the implication of other compounds on this activity. Once formulations were obtained from an ethanolic extract, which contains different compounds [4], they may also contribute to this activity. Interestingly, an increase in the selectivity index (8-fold) was observed when the ASE was incorporated into the nanoemulsion.

**Figure 2:**
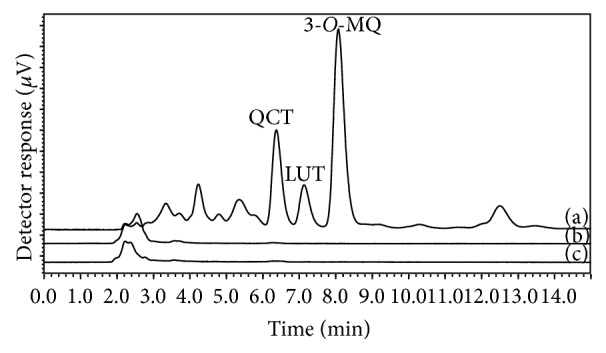
Chromatographic profile of (a)* Achyrocline satureioides* ethanolic extract; (b) porcine ear skin macerate and (c) porcine esophageal mucosa macerate.

Next, we investigated the distribution of the flavonoids into porcine skin and mucosa given that the HSV-1 virus may infect these tissues. The specificity and the recovery of the analytical method were previously determined. As can be seen in Figure 2, the HPLC method is specific, since no interference of the porcine ear skin or esophageal mucosa was detected in the same retention time of QCT, LUT, and 3-*O*-MQ. Moreover, the recovery evaluation (Table 4) demonstrated that the method is also accurate using matrices as porcine ear skin and porcine esophageal mucosa (recovery of 86.52 ± 0.04% to 100.87 ± 0.28%).

In these validated conditions, all flavonoids were found in intact skin (up to 2.28 *µ*g/cm^2^) from ASE-loaded nanoemulsion after 8 h of permeation, mainly in the epidermis (Table 5). No flavonoid was detected in the receptor compartment of Franz diffusion cells. Higher amounts of these flavonoids were detected through the upper (up to 3.0 *µ*g/cm^2^) and deeper (1.8 *µ*g/cm^2^) layers of porcine esophageal mucosa (reaching 4.8 *µ*g per cm^2^ of mucosa), thereby showing a higher permeability of this tissue when compared to the skin [11]. We have used porcine esophageal mucosa instead of buccal mucosa for* in vitro* assays because it offers a larger surface area and has a similar histological composition to that of buccal mucosa [12]. Lastly, it can be noticed that 3-*O*-MQ was also detected in the skin dermis and this flavonoid was able to permeate esophageal mucosa reaching the receptor compartment. Such a result could be related to the higher Log P of 3-*O*-MQ (2.5) when compared to those of QCT and LUT, according to the Bioassay Research Database [13].

Once the treatment of herpetic infections frequently begins following the outbreak of orofacial blisters, experiments were also carried out on previously impaired tissue to gather more information on flavonoid permeation/retention in this condition. The literature reported the use of a tape stripping technique to impair the skin [14, 15]. According to Table 5, a significant increase (*P* < 0.05) in all the three flavonoids was detected in the epidermis when the stratum corneum had been removed, with a 3.2-fold increase in the total amount of flavonoids. Furthermore, these flavonoids were detected in the dermis of the porcine ear skin (3.22 *µ*g/cm^2^). Similarly, the removal of the upper layer of mucosa significantly increased (*P* < 0.05) the retention of QCT, LUT, and 3-*O*-MQ in the deeper layer (by approximately 5 to 7-fold). Moreover, when the mucosa was injured, all flavonoids were detected in the receptor fluid of Franz diffusion cells. Taken together, such results can be related to the lower interaction of the extract compounds with barriers (the corneum stratum and upper layers of the mucosa) due to the physical wound [14] and suggest a higher disposition of the ASE flavonoids during an outbreak of orofacial blisters.

**Figure 3:**
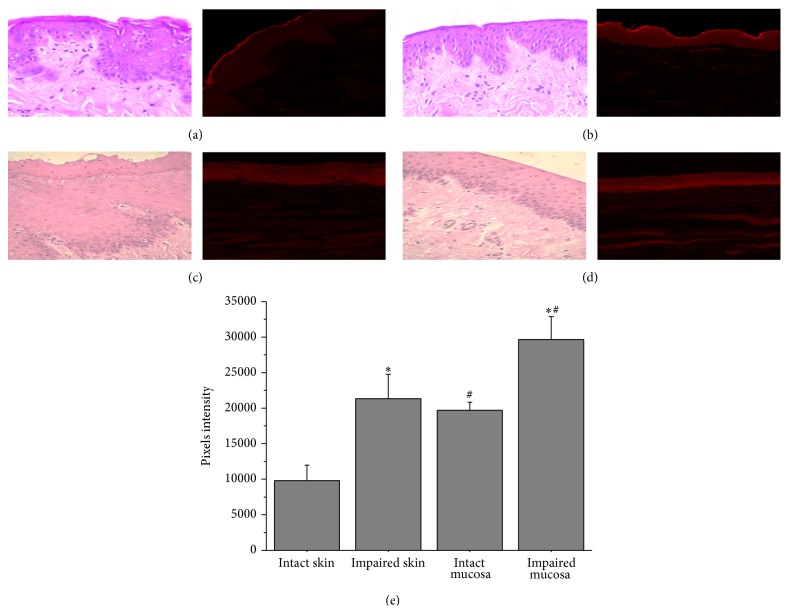
Histological (left line) and fluorescent (right line) images of intact skin (a), impaired skin (b), intact mucosa (c), and impaired mucosa (d). Fluorescent intensity detected for intact or impaired skin and mucosa (e). Significant statistical differences were determined by Tukey's test (*P* < 0.05). Differences between intact and impaired tissues were represented by ∗, while differences between skin and mucosa were represented by #.

A confocal microscopy evaluation was carried out to get more information on increased flavonoid retention in the skin and mucosa. We used Nile Red as a lipophilic dye and fluorescent marker because it is easily soluble in the oil core components.  Figure 3 shows the confocal images and pixel intensity. As it can be seen, a higher amount of fluorescence (19,682 ± 1,152 pixels) was detected in the upper portion of the intact porcine mucosa when compared to the same region of the skin (Tukey's test, *P* < 0.05), suggesting that mucosa enables greater tissue retention as well as higher permeation of bioactive molecules. Furthermore, a significant increase in fluorescence (*P* < 0.05) was detected for impaired skin and esophageal mucosa, which is in agreement with the results concerning flavonoids retention. Impairment of the skin and mucosa was followed by a histological analysis (H&E) which was considered acceptable. In the skin, it was possible to differentiate the stratum corneum, the viable epidermis, and the dermis in intact skin, and the partial removal of the stratum corneum after tape stripping (Figure 3). Intact mucosa consists of three layers of epithelia, the more darkly stained basal layer, the middle layer, with larger and more compact cells, and the superficial stratum, with less compact cells and surrounded by mucus. A complete removal of superficial epithelium was observed in this tissue after gentle scraping.

## 4. Conclusions

We demonstrated that the simultaneous incorporation of ASE flavonoids (QCT, LUT, and 3-*O*-MQ) into a nanoemulsion improved the antiherpes activity of the hydroethanolic extract. Higher flavonoid retention was detected in the porcine ear skin epidermis and in the upper layers of esophageal mucosa. An increase in the retention was observed when these tissues were injured. Overall, such results showed that the ASE-loaded nanoemulsions developed in this study could be considered as a promising formulation for herpes simplex treatment.

## Figures and Tables

**Table 1:** Physicochemical characteristics of QCT- and ASE-loaded nanoemulsions.

Parameter	Blank-NE	QCT-NE	ASE-NE
Droplet size (nm)	207.29 ± 19.11	223.88 ± 7.19	237.35 ± 12.71
Polydispersity index	0.05 ± 0.04	0.07 ± 0.06	0.09 ± 0.04
*ζ*-potential (mV)	−19.48 ± 1.95	−19.19 ± 1.75	−42.45 ± 1.96
Viscosity (cP)	1.66 ± 0.08	1.82 ± 0.07	2.13 ± 0.17

**Table 2:** Flavonoids content in crude ASE and ASE-loaded nanoemulsion.

	QCT	LUT	3-*O*-MQ
ASE (*µ*g/10 mg)	319.00 ± 25.21	202.80 ± 11.12	734.34 ± 68.71
ASE-NE (*µ*g/mL)^a^	307.79 ± 10.23	194.50 ± 7.91	780.35 ± 31.42

^a^1 mL of nanoemulsion contains 1.0% of ASE dried residue (10 mg).

**Table 3:** Antiherpes activity of ASE- and QCT-loaded nanoemulsions and their controls.

	CC_50_ (*µ*g/mL)	IC_50_ (*µ*g/mL)	SI^a^
ASE	15.71 ± 8.61	14.07 ± 3.46	1
ASE-nanoemulsion	11.25 ± 0.48	1.40 ± 0.88^*^	8

QCT	25.34 ± 6.88	7.93 ± 1.53	3
QCT-nanoemulsion	138.52 ± 33.36^*^	9.51 ± 5.98	14

^a^SI: CC_50_/IC_50_; ^*^significant statistical differences were determined by Tukey's test (*P* < 0.05).

**Table 4:** Recovery (%) of QCT, LUT, and 3-*O*-MQ from porcine ear skin and porcine esophageal mucosa matrices.

	Concentration^a^	QCT	LUT	3-*O-*MQ
Skin	Low	99.69 ± 1.62	93.70 ± 2.29	100.87 ± 0.28
	Medium	86.52 ± 0.04	97.82 ± 2.06	98.14 ± 0.37
	High	90.73 ± 0.39	96.07 ± 1.19	96.68 ± 1.90

Mucosa	Low	98.65 ± 0.49	95.65 ± 7.84	100.52 ± 2.57
	Medium	91.12 ± 0.34	96.77 ± 1.92	95.00 ± 1.64
	High	99.89 ± 0.50	98.68 ± 0.89	100.32 ± 1.21

^a^Low = 0.25 *µ*g/mL; medium = 1.0 *µ*g/mL; high = 10.0 *µ*g/mL.

**Table 5:** Flavonoid permeation and retention (*µ*g/cm^2^) using intact and impaired skin and mucosa.

			QCT	LUT	3-*O*-MQ	Total
Skin	Intact	Epidermis	0.35 ± 0.08	0.50 ± 0.05	1.30 ± 0.25	2.20 ± 0.29
		Dermis	ND	ND	0.08 ± 0.02	0.08 ± 0.02
	Impaired	Epidermis	1.04 ± 0.32^*^	1.33 ± 0.26^*^	4.63 ± 0.89^*^	7.03 ± 1.19
		Dermis	0.18 ± 0.01	0.75 ± 0.14	2.41 ± 0.54^*^	3.22 ± 0.73

Mucosa	Intact	Upper layers	0.60 ± 0.08^#^	0.47 ± 0.07	1.97 ± 0.30	3.03 ± 0.44
		Deeper layers	0.33 ± 0.07	0.31 ± 0.06	1.16 ± 0.22^#^	1.80 ± 0.34
		Receptor	ND	ND	0.68 ± 0.14	0.68 ± 0.14
	Impaired	Deeper layers	2.25 ± 0.47^∗#^	1.47 ± 0.34^∗#^	7.47 ± 1.39^∗#^	11.19 ± 2.18
		Receptor	1.69 ± 0.50	2.71 ± 0.53	3.22 ± 1.26^*^	7.62 ± 1.32

ND: not detected; significant statistical differences were determined by Tukey's test (*P* < 0.05); differences between intact and impaired tissues were represented by ∗, while differences between skin and mucosa were represented by #.
